# The Effect of Temperature on Selectivity in the Oscillatory Mode of the Phenylacetylene Oxidative Carbonylation Reaction

**DOI:** 10.1002/cphc.201700359

**Published:** 2017-06-19

**Authors:** Julie Parker, Katarina Novakovic

**Affiliations:** ^1^ School of Chemical Engineering and Advanced Materials Newcastle University, Merz Court Claremont Road Newcastle Upon Tyne UK

**Keywords:** calorimetry, carbonylation, oscillations, reaction mechanisms, selectivity

## Abstract

Reaction temperature plays a major role in product selectivity in the oscillatory mode of the palladium‐catalyzed phenylacetylene oxidative carbonylation reaction. At 40 °C, dimethyl (2Z)‐2‐phenyl‐2‐butenedioate is the major product whereas at 0 °C the major product is 5,5‐dimethoxy‐3‐phenyl‐2(5*H*)‐furanone. The occurrence of oscillations in pH coincides with an increase in the rate of phenylacetylene consumption and associated product formation. Experiments were performed isothermally in a reaction calorimeter to correlate reactant consumption and product formation with the occurrence of pH oscillations and the heat released by the reaction. An increase in the size of the pH drop in a single oscillation correlates with an increase in energy, indicating that this section of a single oscillation relates to reactant consumption. Based on these observations, a reaction pathway responsible for product formation is provided.

Carbonylation reactions are important in synthetic and industrial chemistry as C−C‐bond‐forming reactions that can directly synthesize carbonyl compounds leading to a variety of products.^1^ As the reaction frequently results in several products, costly separation post‐synthesis is needed. Palladium‐catalyzed phenylacetylene oxidative carbonylation (PCPOC) is an extraordinary reaction as it can proceed in an oscillatory mode. PCPOC was found to oscillate in redox potential, pH and the rate of CO/O_2_ gas mixture consumption in a catalytic system (PdI_2_, KI, O_2_, NaOAc in methanol) at 40 °C.^2^ When run in a calorimeter simultaneous oscillations in pH and the rate of heat evolution (Q_r_) were captured^3^ with the total energy release following a staircase function.3b The products reported are shown in Figure 1.3a

**Figure 1:**
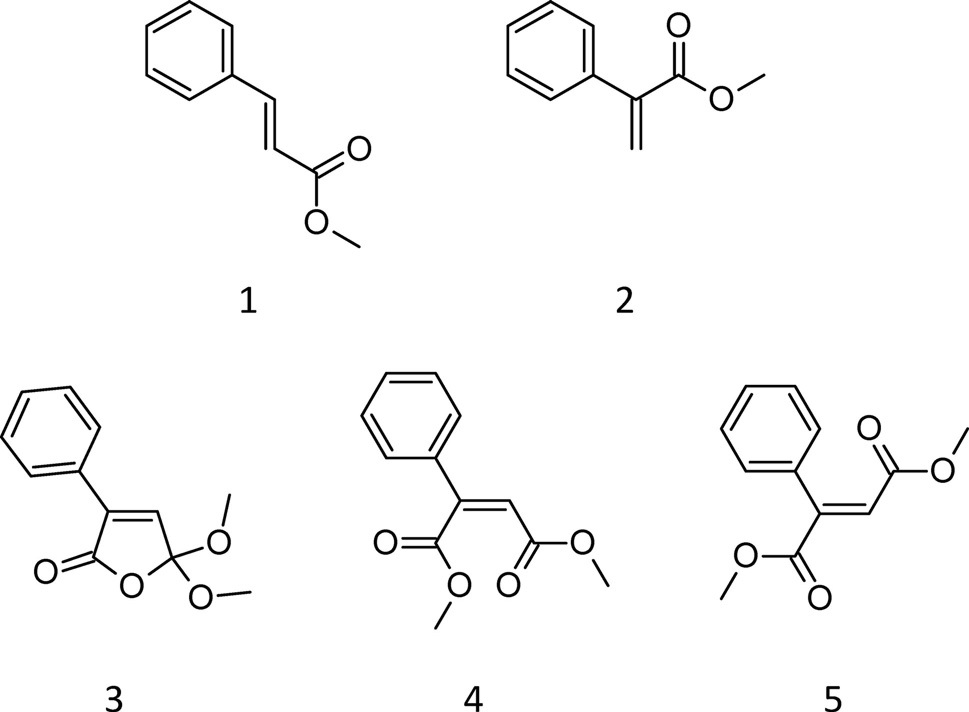
Products of the PCPOC reaction: methyl (*E*)‐cinnamate, **1** (MeCin); methyl atropate, **2** (MeAt); 5,5‐dimethoxy‐3‐phenyl‐2(5*H*)‐furanone, **3** (DMO); dimethyl (2Z)‐2‐phenyl‐2‐butenedioate, **4** (Z‐isomer) and dimethyl (2E)‐2‐phenyl‐2‐butenedioate, **5** (E‐isomer).3a

Fascinatingly, at 40 °C, the occurrence of pH oscillations was reported to affect product selectivity.3c When operating in an oscillatory pH regime, product formation was suppressed until oscillations occurred, followed by selective formation of **4**. When operating in a non‐oscillatory pH regime, selectivity was poor with the main products being **1, 2** and **4**. For the same initial conditions, oscillatory and non‐oscillatory regimes were achieved by changing the amount of PdI_2_ catalyst. The influence of reaction temperature on the period and amplitude of pH oscillations during the PCPOC reaction was also captured.3d Isothermal experiments performed over the temperature range 10–50 °C demonstrated the existence of oscillations in the range 10–40 °C, with a decrease in reaction temperature resulting in an increase in the period and amplitude of the pH oscillations. However, the associated effect of reducing reaction temperature on product formation during the PCPOC reaction in oscillatory mode was not investigated.

The study presented here investigates the effect of the oscillatory mode of the PCPOC reaction on the dynamics of product formation over the temperature range of 0–40 °C. The experiments were conducted in a reaction calorimeter under isothermal conditions at 0, 10, 20, 30 and 40 °C while monitoring pH, reaction heat and the dynamics of reactant consumption and product formation using the previously published method (see Supporting Information, SI).3a, 4 Figure 2 a –e shows the product and reactant concentration profiles along with the recorded pH at each temperature. A summary of the oscillatory characteristics is given in Table S1 in the SI.

**Figure 2:**
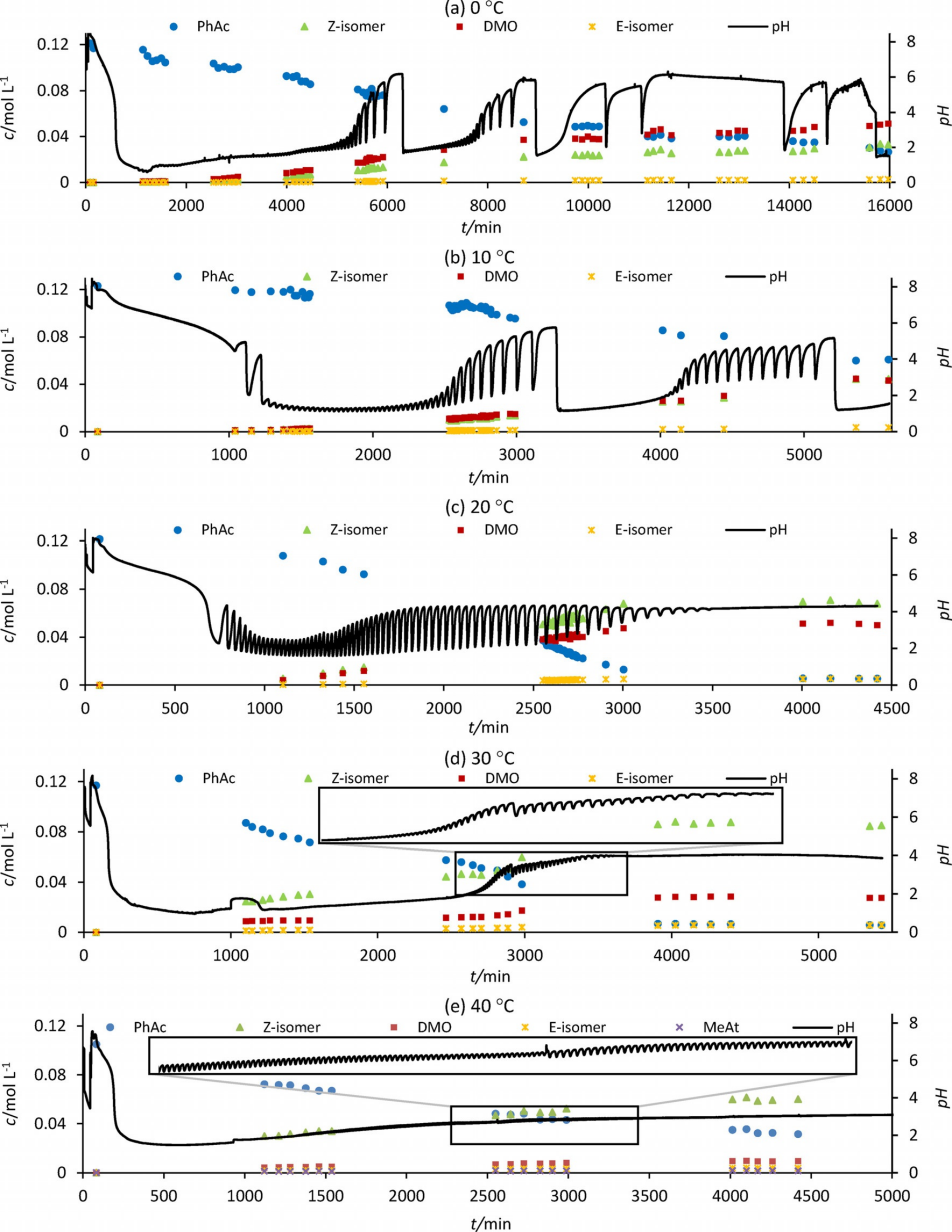
pH^4^ and concentrations of phenylacetylene (PhAc) and products during the PCPOC reaction: a) 0 °C; b) 10 °C; c) 20 °C; d) 30 °C; e) 40 °C.

Reproducibility of the oscillations in pH and key characteristics of the pH profiles at each temperature have been reported previously.3d Although batch oscillators are difficult to align fully at specific time points, good reproducibility is demonstrated in the overall pH behavior (see SI, Figures S1–S4). Long lasting pH oscillations without further addition of substrate are noted in Figure 2: a feature exclusive to this oscillatory system. The products detected at all temperatures were: DMO, **3**; Z‐isomer, **4** and E‐isomer, **5** (Figure 1).3a Small amounts of MeAt, **2**, were detected at 40 °C while MeCin, **1**, was not detected.

Figure 2 and Table S1 (SI) show that the period and amplitude of the pH oscillations increase as temperature decreases, in agreement with previous studies at 10–40 °C.3d The trend continues further to 0 °C. The duration of oscillations at 0–30 °C correlates with reaction temperature, with the shortest duration oscillations occurring at 30 °C. The duration of oscillations at 40 °C is longer than expected and significantly longer than that reported earlier for this reaction temperature: 4389 min here compared to a maximum of 760 min previously.3c The oscillations at 40 °C were also much smaller in amplitude than previously reported which is why they lasted longer.3b–3d At all temperatures, the rate of PhAc consumption and product formation accelerated with the occurrence of oscillations. A comparison of reactant conversion and the formation of individual products is given in Figure 3 a–d.

**Figure 3:**
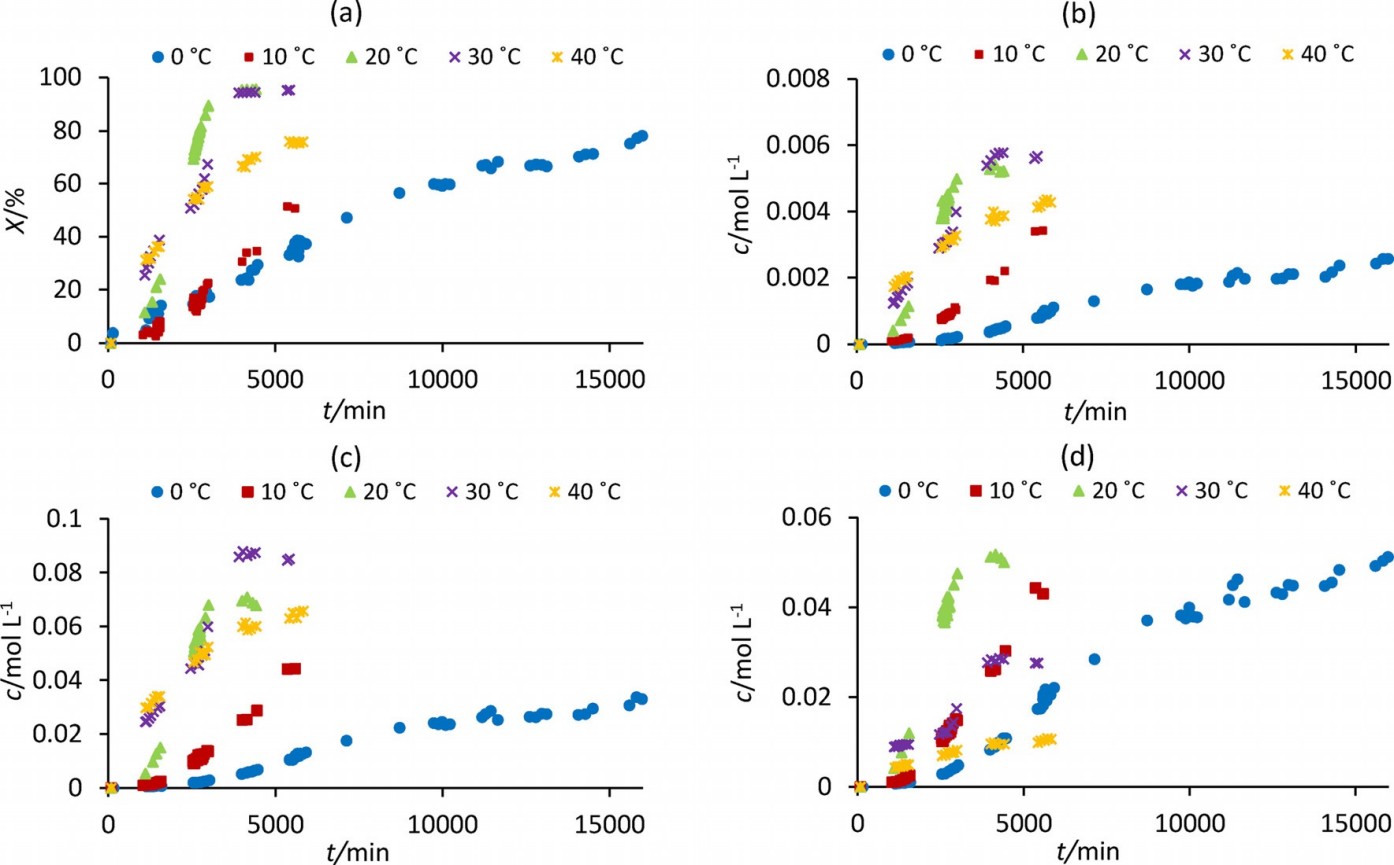
PhAc conversion and product concentrations during the PCPOC reaction at temperatures of 0–40 °C: a) PhAc; b) **5**; c) **4**; d) **3**.

From Figure 3 the conversion of PhAc to products recorded at 0–20 °C follows kinetic laws: conversions increase as the temperature increases. This is less apparent at 30 °C and is not followed at 40 °C, suggesting thermodynamic reaction control. The carbonylation of PhAc has been shown to result in the formation of **4** as the major product at temperatures of 25–80 °C under a pressurized atmosphere containing CO and air although no mention is made of whether the reaction was conducted in an oscillatory mode.1c We found that, in oscillatory mode, the major product depends on the reaction temperature. As temperature increases, formation of **4** increases, while at lower temperatures the formation of **3** is favored with **3** being the major product at 0 and 10 °C. The ratio, *r*, of **3** and **4** remains constant during the reaction (Figure 4 a) and decreases with increasing temperature. This suggests the activation energies of the two pathways leading to formation of these two products differ, resulting in reaction temperature dictating which process will dominate, kinetically or thermodynamically controlled.^5^ Formation of **3** is the faster reaction, that is, under kinetic control, and dominates at lower temperatures whilst **4** is the more thermodynamically stable product (i.e. under thermodynamic control) and dominates at higher temperatures. Figure 4 b shows the ratio of **5** and **4** at 0–20 °C is similar and slightly higher than that at 30 and 40 °C once appreciable amounts of both isomers are formed suggesting their formation follows a related reaction pathway.

**Figure 4:**
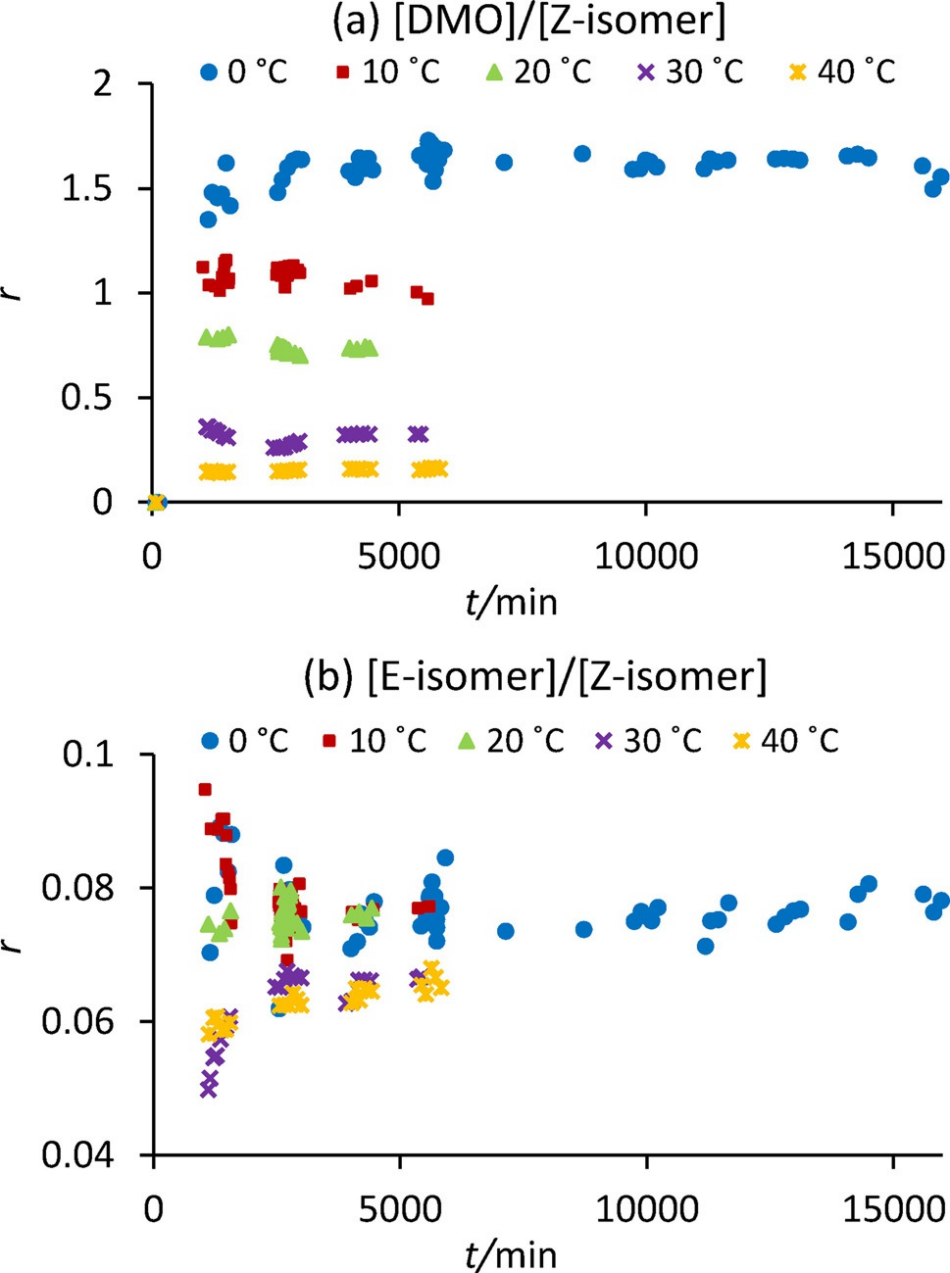
Product ratios during the oscillatory PCPOC reaction at 0–40 °C: a) **3**/**4**; b) **5**/**4**.

The lengthy experiments incurred evaporative loss of solvent affecting the accuracy of measurements and making it challenging to capture trends in energy release for the full duration of the experiments. This particularly affected the run at 40 °C where methanol was added regularly to compensate for this loss. In addition, at 40 °C the period and amplitude of oscillations was very small reflecting small energy changes which are difficult to measure accurately. Moreover, reducing the reaction temperature slows down the rate of the reaction leading to a corresponding reduction in the rate at which heat is produced. Thus, it was only possible to observe the energy release profile over a large section of the reaction at 30 °C (Figure 5). Figure 5 a shows heater power directly correlates with the pH oscillations. As a result, the heat the reaction produces (Q_r_) is released in pulses, causing the total energy release to increase stepwise (Figure 5 b –d).

**Figure 5:**
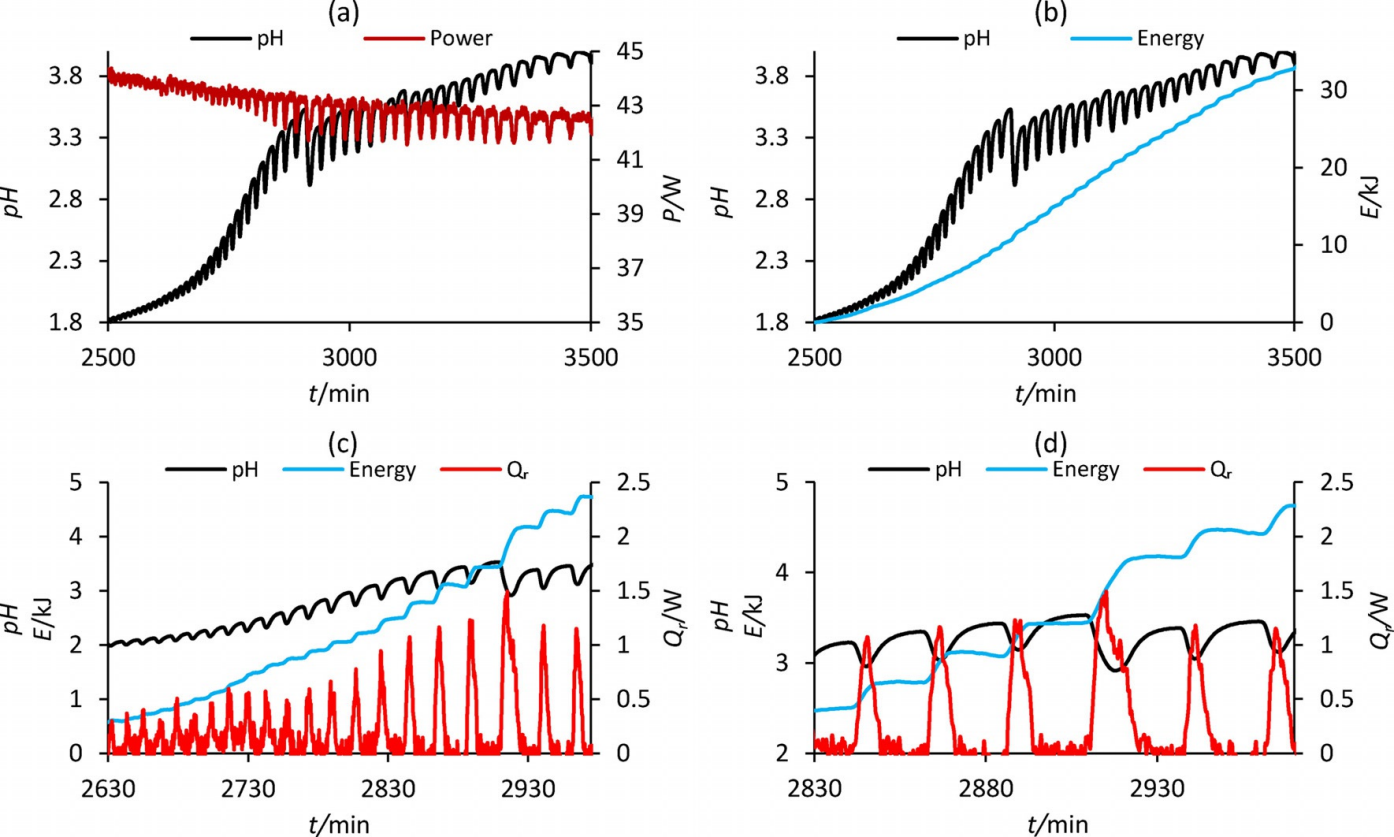
Release of energy in the PCPOC reaction at 30 °C: a) oscillations in heater power in‐phase with pH oscillations; b) stepwise release of energy during pH oscillations; c) amplitude of pH and Q_r_ oscillations increases simultaneously and pH is correlated with energy release; d) pH drop at 2910 min is associated with a larger step increase in energy.

Closer examination of proximate oscillations at 30 °C shows that the amount of energy released increases as the size of the pH drop increases (Figure 5 c). Figures 5 c and 5 d show the large drop in pH at approximately 2910 min is accompanied by a release of 0.7 kJ of energy compared to the 0.3 kJ of energy released during the previous and subsequent oscillations. The stepwise release of energy that occurs at 30 °C is also evident at 0, 10, 20, and 40 °C when shorter sections of the reaction are examined, verifying the relationship between changes in pH and energy release (Figure 6 a –d). It can be concluded that the section of a single oscillation when the pH drops relates to reactant consumption inducing energy release. This accelerated pH fall is therefore associated with the acceleration in reaction rate and consequently product formation.

**Figure 6:**
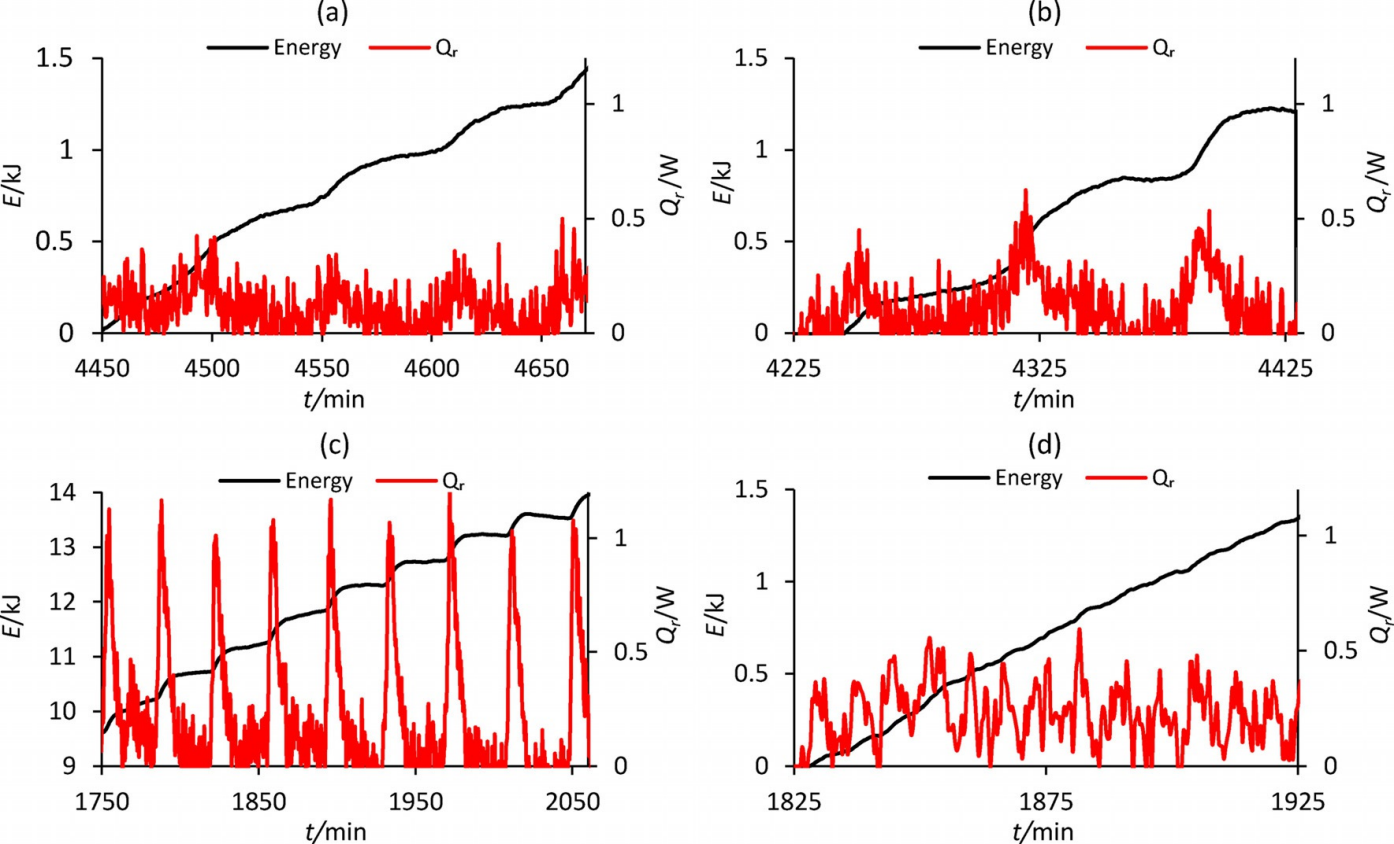
Stepwise release of energy during the PCPOC reaction at a) 0 °C, b) 10 °C, c) 20 °C and d) 40 °C.

A number of mechanisms for palladium‐catalyzed carbonylation of alkynes have been proposed.1c, 2, 6 Combined with the observations in this study, a reaction pathway that can explain the products detected under the reaction conditions described here is given in Scheme 1.

**Scheme 1:**
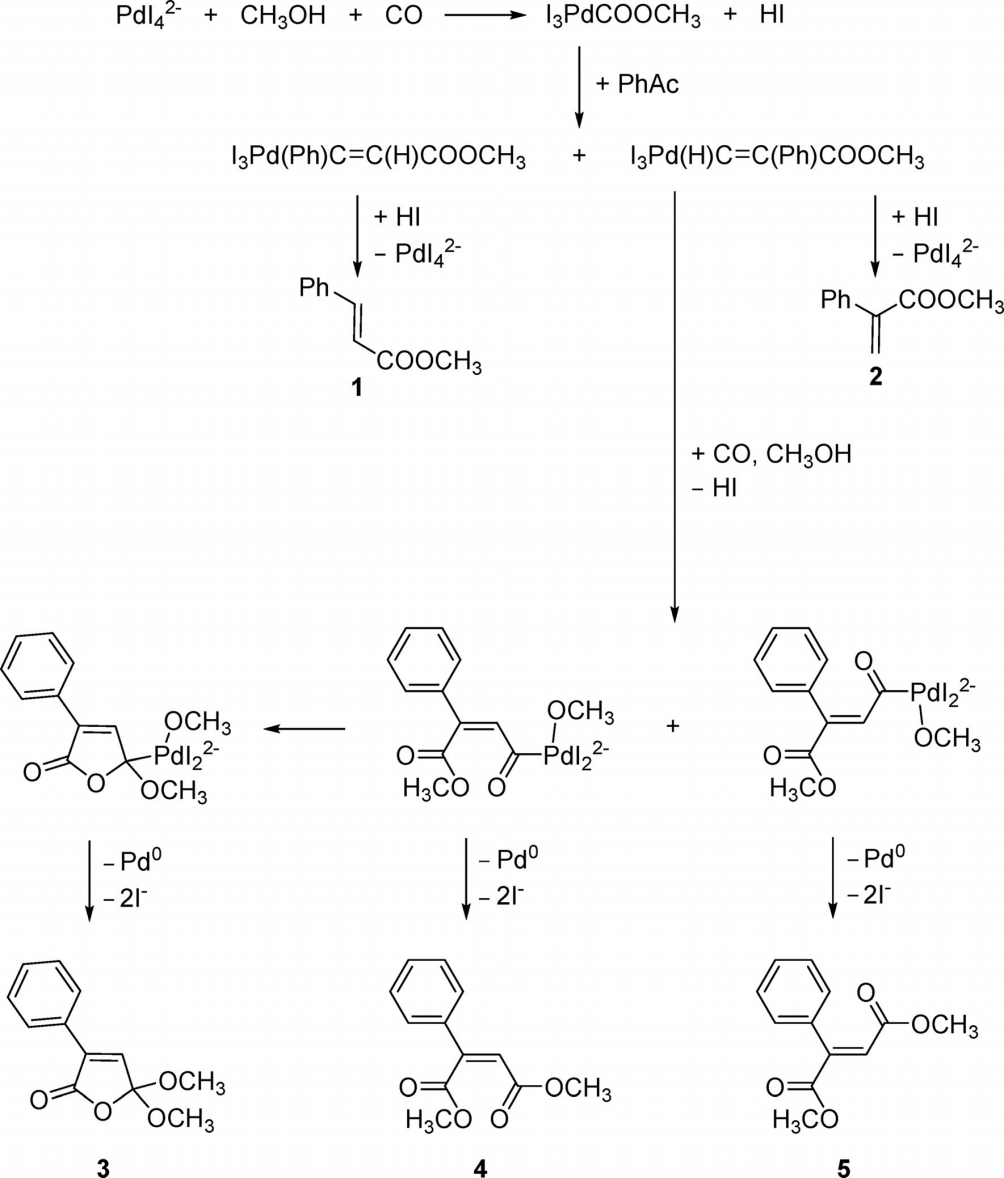
Reaction pathway.

KI and PdI_2_ react to form K_2_PdI_4_ which then goes on to react with methanol and CO to form I_3_PdCOOCH_3_ and HI.1c, 6b This is supported by experiments which confirm a drop in pH occurs when PdI_2_, KI and methanol are purged with CO indicating the formation of HI.^7^ The next stage is the insertion of phenylacetylene into the Pd−C bond in I_3_PdCOOCH_3_.1c, 6b Depending on the orientation of the PhAc insertion this could lead to either product **1** or **2**. This is supported by the detection of **2** in this study at 40 °C and **1** in previous studies, although in trace amounts.^8^ In previous studies **1** appeared to behave as an intermediate, being produced and consumed. However, subsequent experiments in which **1** was used as the starting material instead of PhAc failed to produce the diester products indicating that **1** is not a reaction intermediate.^8^ As only trace levels of **2** were detected in this study at 40 °C it suggests that monoester formation under these conditions is unfavorable. As the major products of this reaction are the diester products, the reaction of I_3_Pd(H)C=C(Ph)COOCH_3_ with further CO and MeOH is required. This reaction occurs with the release of HI which is supported by the observed drop in pH and associated energy pulse that occurs within each pH oscillation. This second reaction with CO and MeOH results in 2 isomeric intermediates which produce **4** and **5** with the release of Pd^0^ and I^−^. The palladium is then recycled reforming [PdI_4_]^2−^ and consuming HI in the process.^9^ This is borne out by the observations of oscillations in turbidity which have previously been reported for this system.^4^ The isomeric intermediate leading to **4** is able to undergo a cyclisation process which ultimately results in the formation of **3** accompanied by the release of Pd^0^ and I^−^.1c, 6b

In conclusion, this study has shown that reaction temperature can be used to tune selectivity of the PCPOC reaction when operated in oscillatory mode. Under otherwise the same conditions, at 0 °C **3** is the major product while at 40 °C the major product is **4**. The stepwise release of energy that occurs during the reaction has been shown to correlate with the pH fall within each oscillation. A plausible reaction pathway explaining the observed product distribution has been given.

## Conflict of interest

*The authors declare no conflict of interest*.

## Supporting information

As a service to our authors and readers, this journal provides supporting information supplied by the authors. Such materials are peer reviewed and may be re‐organized for online delivery, but are not copy‐edited or typeset. Technical support issues arising from supporting information (other than missing files) should be addressed to the authors.

Supplementary
