# Supplementary material for: The Effect of Temperature on Selectivity in the Oscillatory Mode of the Phenylacetylene Oxidative Carbonylation Reaction
Source: Chemphyschem. 2017 Jun 19;18(15):1981–6. doi: 10.1002/cphc.201700359 (PMC5575551; doi:10.1002/cphc.201700359)
Supplement: Supplementary file 1 — Supplementary [file CPHC-18-1981-s001.pdf]

CHEMPHYSCHEM

Supporting Information

The Effect of Temperature on Selectivity in the Oscillatory Mode of the Phenylacetylene Oxidative Carbonylation Reaction

Julie Parker* and Katarina Novakovic[a]

cphc_201700359_sm_miscellaneous_information.pdf

Supporting Information

Contents

Experimental Section 1

Additional Results 2

References 4

Experimental Section

All experiments were carried out in an HEL Simular reaction calorimeter which consists of a 1 L double-jacketed glass reactor having an outer vacuum jacket and an inner oil jacket. The reactor has a 150 W Hastelloy C276 internal electrical heater, 0-600 rpm PTFE pitched blade downflow stirrer, a Pt100 temperature probe and a glass combined pH electrode. The oil jacket temperature is controlled by a Julabo FP50HD refrigerating and heating circulator. There are two mass flow controllers: one controls the flow of air and the other controls the flow of CO. The experiments were performed at temperatures from 0-40 °C using power compensation calorimetry. The compensation drop was set at 20 °C i.e. the oil jacket temperature was maintained at a value 20 °C below the desired process temperature while the internal electrical heater was set to achieve the difference and maintain the temperature at the desired process value. Therefore, the electrical heater power varied throughout the chemical reaction process, compensating for the changes in process heat load. Quantification of products was achieved by adding naphthalene to the reactor vessel as an internal standard for the GCMS analysis. The addition of naphthalene allowed any changes in volume due to the evaporative loss of solvent to be accounted for and has been shown to have no effect on the reaction or the pH oscillations.[1]

All chemicals were purchased from Sigma-Aldrich and used as received. Initially the reactor was filled with 400 mL of HPLC grade methanol (Sigma-Aldrich 34860) and heating commenced. The PdI_2 (434 mg, 1.2 mmol, Sigma-Aldrich 203963) was added after 5 min and the mixture was stirred for 35 min with the stirrer speed set at 250 rpm. After 35 min, 113 mg NaOAc (1.4 mmol, Sigma-Aldrich 241245) were added followed by 37.393 g of KI (225 mmol, Sigma-Aldrich 221945) and 30 mL MeOH. The stirrer speed was increased to 350 rpm to aid the dissolution of the KI. After 10 min, 1.154 g of naphthalene (Np, 9.0 mmol, internal standard) was added followed by 20 mL MeOH and the mixture was stirred for a further 5 min before purging with CO (50 $mLmin^{-1}$) and air (50 $mLmin^{-1}$) commenced. After 20 min of purging, phenylacetylene (PhAc, 6.2 ml, 98%, Sigma-Aldrich 117706) was added straight from the fridge. After allowing 2 min for mixing, the first sample was taken and sampling continued periodically throughout the experiments. All samples were filtered over silica prior to being diluted 1:9 with methanol. The samples were analysed by a Varian Saturn 2200 GCMS fitted with a VF-5ms column (30 m). The method was as follows: injector temperature 150 °C; helium flow rate 1 $mLmin^{-1}$; oven temperature 100-195 °C over 35 min in 5 steps.

Table 1. A summary of the main features of the pH oscillations at 0-40 °C.

Temperature (°C)	Time of onset (min)	Duration (min)	Period (min)		pH at onset (pH units)	pH range (pH units)	Amplitude (pH units)	
			Max	Min			Max	Min
0	990	16000[a]	1375	47	0.82	0.59-6.13	3.61	0.04
10	1042	4201[a]	189	27	4.44	1.13-5.75	3.18	0.012
20	744	2737	71	22	2.27	1.58-4.35	2.35	0.071
30	2421[b]	1190	45	6	1.74	1.74-4.01	0.614	0.003
40	1270	4389	10	5	1.93	1.93-3.14	0.164	0.002

[a]Experiment was stopped before oscillations had ceased.

[b]Some oscillations started at 270 min but were not sustained.

Additional Results

Repeat experiments were conducted at 10-40 °C and the pH results are shown in Figures 1-4. Although the data do not align at specific time points it is evident that the overall pH behavior at each temperature is consistent. The difference in the induction period at each temperature is likely due to the difference in the granularity of the PdI_2 catalyst which has been shown to vary from a fine powder (µm) to 1-2 mm chunks and affects the rate at which the catalyst dissolves.[2]

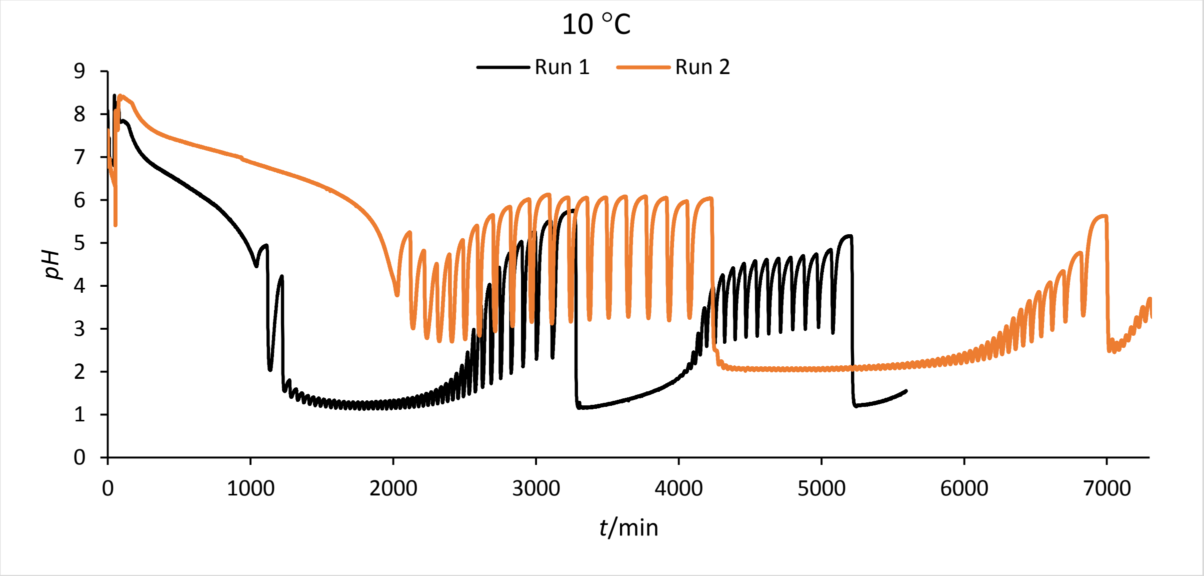

Figure 1: PCPOC reaction conducted in an HEL Simular reaction calorimeter at 10 °C: PdI_2 = 1.2 mmol; MeOH = 450 mL; KI = 225 mmol; NaOAc = 1.4 mmol; PhAc = 6.2 mL; CO = 50 $mLmin^{-1}$; air = 50 $mLmin^{-1}$; Np = 9.0 mmol.

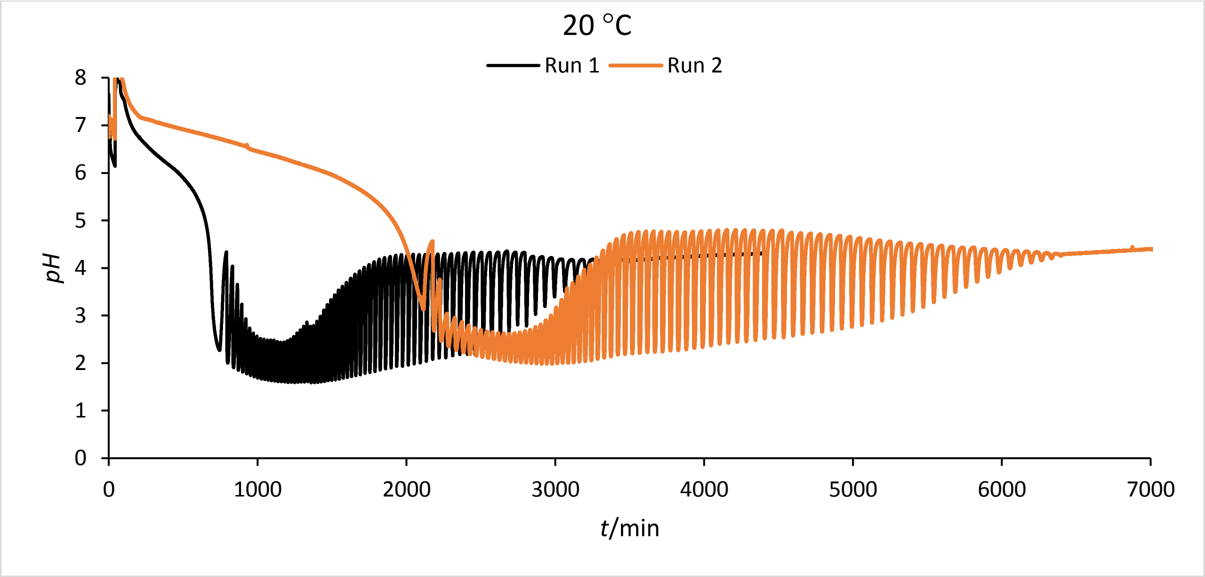

Figure 2: PCPOC reaction conducted in an HEL Simular reaction calorimeter at 20 °C: PdI_2 = 1.2 mmol; MeOH = 450 mL; KI = 225 mmol; NaOAc = 1.4 mmol; PhAc = 6.2 mL; CO = 50 $mLmin^{-1}$; air = 50 $mLmin^{-1}$; Np = 9.0 mmol.

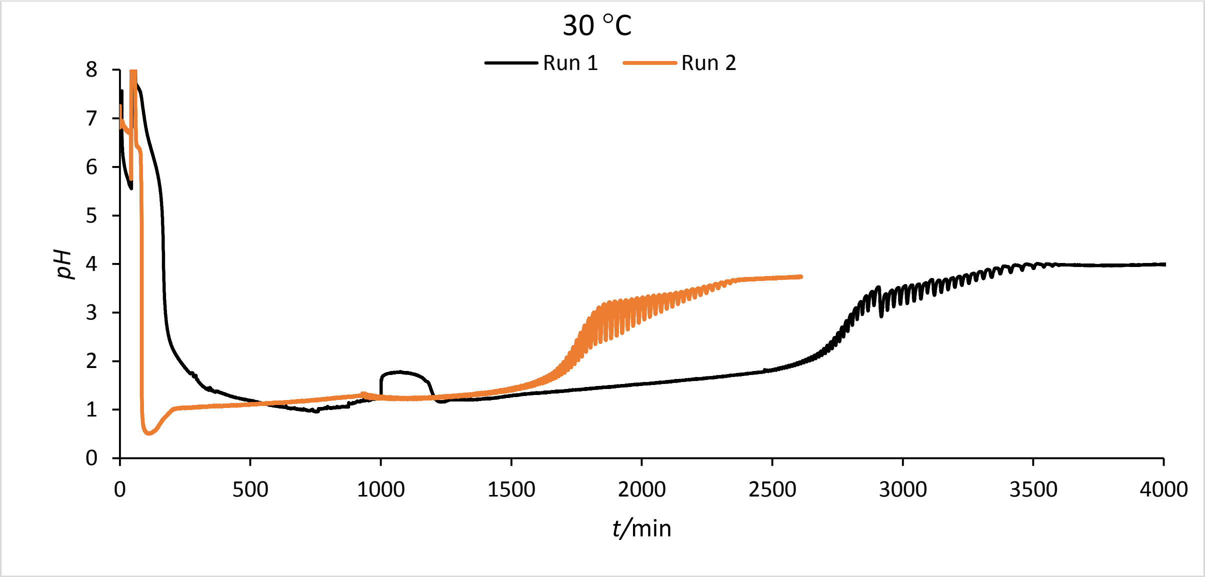

Figure 3: PCPOC reaction conducted in an HEL Simular reaction calorimeter at 30 °C: PdI_2 = 1.2 mmol; MeOH = 450 mL; KI = 225 mmol; NaOAc = 1.4 mmol; PhAc = 6.2 mL; CO = 50 $mLmin^{-1}$; air = 50 $mLmin^{-1}$; Np = 9.0 mmol.

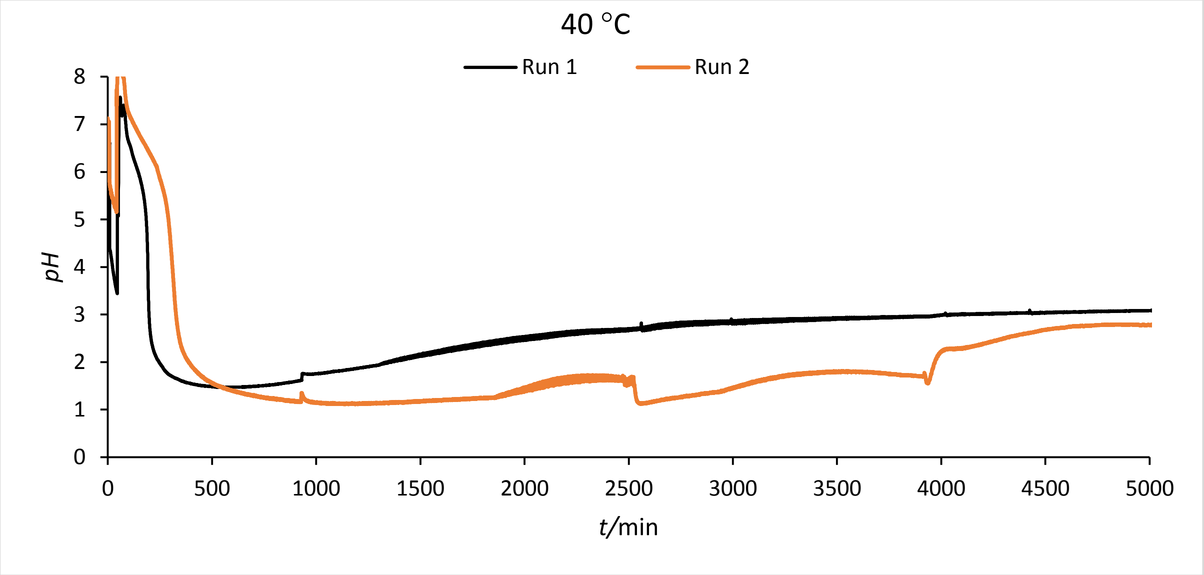

Figure 4: PCPOC reaction conducted in an HEL Simular reaction calorimeter at 40 °C: PdI_2 = 1.2 mmol; MeOH = 450 mL; KI = 225 mmol; NaOAc = 1.4 mmol; PhAc = 6.2 mL; CO = 50 $mLmin^{-1}$; air = 50 $mLmin^{-1}$; Np = 9.0 mmol.

The disturbances in pH in Figure 4 Run 2 at 2470 min and 3919 min coincide with the addition of methanol (100 mL and 130 mL respectively) to the system to compensate for evaporative loss of the solvent.

References

[1] J. Parker, Doctoral Thesis, Newcastle University, Newcastle Upon Tyne, UK thesis, Newcastle University (Newcastle Upon Tyne, UK), **2016**.

[2] K. Novakovic, C. Grosjean, S. K. Scott, A. Whiting, M. J. Willis, A. R. Wright, *PCCP* **2008**, 749-753.
